# Interneuronal GluK1 kainate receptors control maturation of GABAergic transmission and network synchrony in the hippocampus

**DOI:** 10.1186/s13041-023-01035-9

**Published:** 2023-05-20

**Authors:** Simo Ojanen, Tatiana Kuznetsova, Zoia Kharybina, Vootele Voikar, Sari E. Lauri, Tomi Taira

**Affiliations:** 1 Department of Veterinary Biosciences, Faculty of Veterinary Medicine, University of Helsinki, Helsinki, Finland; 2 HiLife Neuroscience Center, University of Helsinki, Helsinki, Finland; 3 Molecular and Integrative Biosciences Research Program, University of Helsinki, Helsinki, Finland

**Keywords:** Glutamate receptor, Kainate receptor, GABAergic interneuron, Hippocampus, Network synchronization, Gamma oscillation, Cognitive flexibility

## Abstract

Kainate type glutamate receptors (KARs) are strongly expressed in GABAergic interneurons and have the capability of modulating their functions via ionotropic and G-protein coupled mechanisms. GABAergic interneurons are critical for generation of coordinated network activity in both neonatal and adult brain, yet the role of interneuronal KARs in network synchronization remains unclear. Here, we show that GABAergic neurotransmission and spontaneous network activity is perturbed in the hippocampus of neonatal mice lacking GluK1 KARs selectively in GABAergic neurons. Endogenous activity of interneuronal GluK1 KARs maintains the frequency and duration of spontaneous neonatal network bursts and restrains their propagation through the hippocampal network. In adult male mice, the absence of GluK1 in GABAergic neurons led to stronger hippocampal gamma oscillations and enhanced theta-gamma cross frequency coupling, coinciding with faster spatial relearning in the Barnes maze. In females, loss of interneuronal GluK1 resulted in shorter sharp wave ripple oscillations and slightly impaired abilities in flexible sequencing task. In addition, ablation of interneuronal GluK1 resulted in lower general activity and novel object avoidance, while causing only minor anxiety phenotype. These data indicate a critical role for GluK1 containing KARs in GABAergic interneurons in regulation of physiological network dynamics in the hippocampus at different stages of development.

**Supplementary Information:**

The online version contains supplementary material available at 10.1186/s13041-023-01035-9.

## Introduction

Balanced interplay between GABAergic and glutamatergic transmission is important for generation of coordinated network activity both during development and in the adult brain [1–4]. In the immature circuitry, the high-frequency oscillatory components of spontaneous network activity provide the functional templates for activity-dependent synaptogenesis [2, 5–7, reviewed in [8]] and constrain apoptosis [8, 9], thereby shaping the development and fine-tuning of the network connectivity. In the adult circuitry, GABAergic activity maintains and paces the rhythmic oscillations suggested to underlie various cognitive and motor functions [10].

Kainate receptors (KARs) are ionotropic glutamate receptors, also capable of metabotropic functions, that can profoundly influence the balance between inhibition and excitation in neuronal networks [11–14]. They consist of five subunits, GluK1—GluK5, encoded by genes *Grik1—Grik5* [12]. GluK1 in particular, has an interesting developmental expression pattern in the hippocampus, being expressed in both principal neurons and GABAergic interneurons during the first postnatal weeks [15], after which its expression is mainly confined to interneurons [16–18]. The early GluK1 activity in principal neurons plays a crucial role in regulating glutamate release, synaptic plasticity and activity-dependent development of functional neural networks both in the hippocampus and in the amygdala [19–21]; however, the role of interneuronal GluK1 in development of behaviorally relevant neural circuits is less clear.

Pharmacological studies have indicated that activation of GluK1 KARs strongly recruits spontaneous GABAergic activity by direct ionotropic depolarization of interneurons (e.g. [22–24]). In addition, endogenous activity of GluK1 subunit containing KARs in the immature hippocampus maintains high excitability of interneurons, typical for early development, by regulating the function of calcium-activated potassium channels (SK channels) in a G-protein dependent manner [42]. Both mechanisms result in increased activation of GABAergic neurons, suggesting that the physiological role of interneuronal GluK1 KARs is to act as a feedback mechanism to maintain the network excitability in a critical range during developmental and activity-dependent fluctuations in the levels of endogenous glutamate [11]. However, activation of GluK1 KARs can also result in depression of evoked GABAergic transmission via G-protein dependent inhibition of GABA release [23, 25], particularly at CCK/CB1 expressing interneurons [26], suggesting that interneuronal KARs might have more complex roles in shaping the network activity patterns towards adulthood.

These data indicate that GluK1 KARs are closely involved in functional regulation of the GABAergic network in the early postnatal hippocampus and suggest that its malfunction might influence adult oscillatory network activity and associated behaviors. However, surprisingly little data exists on the roles of KARs in regulation of physiological circuit activity and brain oscillations. Here we take the advantage of a mutant mouse line in which GluK1 expression has been selectively ablated in GABAergic interneurons, to understand the significance of interneuronal GluK1 for neonatal neurotransmission and network synchronization. Furthermore, the consequences on adult hippocampal activity such as theta and gamma oscillations and sharp wave ripples are investigated in parallel with hippocampus dependent behaviors.

## Results

### Loss of GluK1 expression in the interneurons delays development of GABAergic synaptic transmission

**Fig. 1:**
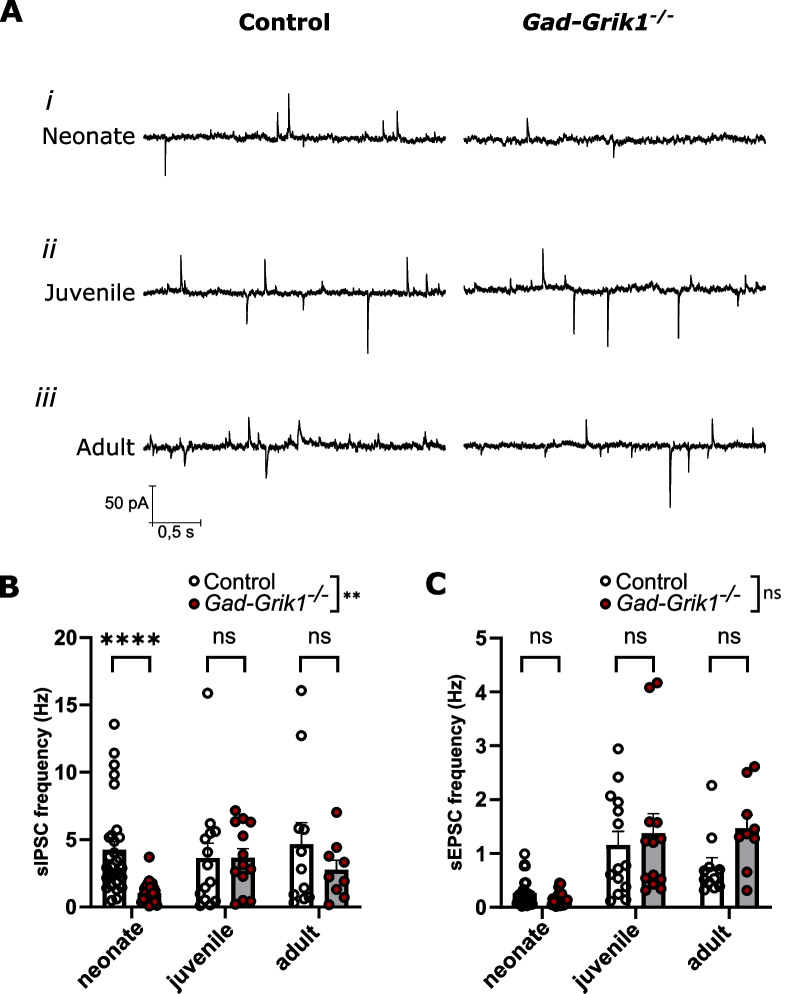
Ablation of GluK1 in GABAergic interneurons attenuates GABAergic synaptic activity in the neonatal but not juvenile or adult CA3. **A** Example traces of whole-cell patch-clamp recordings from neonatal (*i*), juvenile (*ii*) and adult (*iii*) control (left) and *Gad-Grik1*^−/−^ (right) mice with sIPSCs appearing as outward and sEPSCs as inward currents. **B**, **C** Basal frequency of sIPSCs (**B**) and sEPSCs (**C**) in pyramidal CA3 cells from acute control and *Gad-Grik1*^−/−^ mice slices across different age groups (neonatal: n = 30 (21) and 24 (19); juvenile: n = 14 (12) and 13(11); adult: n = 11 (10) and 9 (9), for control and *Gad-Grik1*^−/−^ respectively; n refers to number of cells, followed by number of animals in parenthesis. Bars represent mean ± SEM. Frequencies were compared by 2-way ANOVA with multiple Mann–Whitney test as a post-hock to detect differences in genotype for each age group. **** p < 0.000001 (Mann–Whitney test)

To monitor how loss of GluK1 expression selectively in GABAergic neurons affects synaptic transmission at different stages of development, we performed whole-cell patch clamp recordings from pyramidal cells located in the CA3 region in acute slices from control mice, with floxed *Grik1* (*Grik1*^*tmc1/tm1c*^), and mutant mice lacking GluK1 expression in the GABAergic interneurons (*Gad2*-*Grik1*^*tm1d/tm1d*^). From here on, these mice are referred to as control and *Gad-Grik1*^−/−^, respectively. To be able to look at GABAergic and glutamatergic activity simultaneously, we used an intracellular solution with low chloride (2 mM) concentration in order to shift GABA-A reversal potential [22] and clamped the membrane potential at − 50 mV. Under these conditions, spontaneous inhibitory GABA-A receptor mediated events (sIPSCs) appeared as an outward currents and excitatory glutamatergic events (sEPSCs) as inward currents (Fig. 1A). We did not observe any difference in sEPSC frequency between control and *Gad-Grik1*^−/−^ groups across different developmental stages (2-way ANOVA, p = 0.3; Fig. 1C). In both genotypes, sEPSCs were very rare in neonatal mice (0.265 ± 0.046 Hz and 0.153 ± 0.026 Hz; Fig. 1A*i*), but became more frequent in juvenile (1.161 ± 0.250 Hz and 1.377 ± 0.361 Hz; Fig. 1A*ii*) and adult groups (0.752 ± 0.171 Hz and 1.469 ± 0.250 Hz, Fig. 1A*iii*, for control and *Gad-Grik1*^−/−^, respectively; age effect p < 0.0001; Fig. 1C). On the contrary, the frequency of sIPSCs was different between control and *Gad-Grik1*^−/−^ mice (2-way ANOVA, genotype effect p = 0.002), in particular in the neonatal group, where it was dramatically lower in the *Gad-Grik1*^−/−^ (1.036 ± 0.161 Hz) as compared to the control mice (4.243 ± 0.617 Hz; p < 0.000001, Mann–Whitney test; Fig. 1A*i*, B). The reduced sIPSC frequency in the mutants did not persist later in life and was comparable to that in controls in both juvenile (3.655 ± 0.689 Hz, *Gad*-*Grik1*^−/−^ vs 3.636 ± 1.113 Hz, control) and adult (2.762 ± 0.711 Hz in *Gad*-*Grik1*^−/−^ vs 4.666 ± 1.586 Hz in control; Mann–Whitney test) mice (Fig. 1A*ii, iii*, B). We did not observe any significant differences between genotypes in sIPSC or sEPSC amplitudes (Additional file 1: Fig. S1A). Taken together, these results indicate that the absence of GluK1 in the GABAergic interneurons results in impaired GABAergic transmission to CA3 pyramidal neurons in the neonatal hippocampus, but its effect is developmentally restricted and levels out later in life.

### GABAergic and glutamatergic synaptic transmission is regulated by distinct subpopulations of GluK1 KARs in the neonatal hippocampus

To further understand the cell-type specific roles of GluK1 in regulation of synaptic transmission at different stages of development, we tested the effects of GluK1-selective agonist ATPA and antagonist ACET on spontaneous synaptic activity in CA3 pyramidal neurons from control and *Gad-Grik1*^−/−^ mice.

**Fig. 2:**
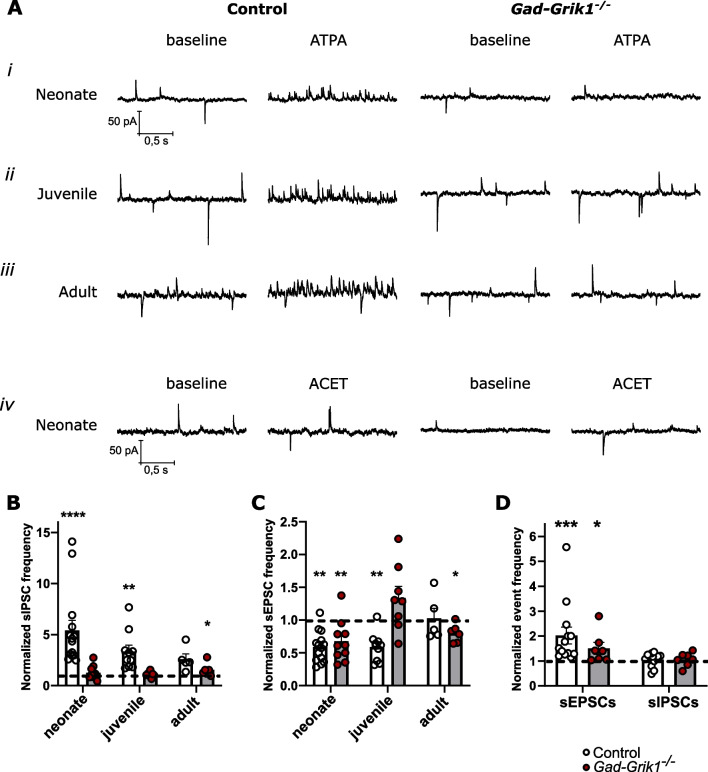
Pharmacological characterization of spontaneous synaptic activity in *Gad-Grik1*^−/−^ mice. **A** Example traces of recordings from neonatal (*i* and *iv*), juvenile (*ii*) and adult (*iii*) control (left columns) and *Gad-Grik1*^−/−^ (right columns) slices, before (baseline) and during ATPA (*i*, *ii*, *iii*) or ACET (*iv*) application. **B** Pooled data illustrating the effect of ATPA (1 µM) on sIPSC frequency in CA3 pyramidal cells from acute control and *Gad-Grik1*^−/−^ slices at different stages of development (neonatal: n = 14 (10) and 10 (10); juvenile: n = 10 (10) and 8 (7); adult: n = 5 (5) and 6 (6), for control and *Gad-Grik1*^−/−^, respectively). **C** Effect of ATPA on sEPSC frequency, for the same cells as in **B**. **D** Effect of ACET (200 nM) on the frequency of sEPSCs and sIPSCs in neonatal control and *Gad-Grik1*^−/−^ slices (n = 13 (N = 10) and 7 (6), for control and *Gad-Grik1*^−/−^, respectively). Bars represent mean ± SEM. Frequency of events is normalized to the baseline (dashed line). Activity during ATPA / ACET application is compared to the baseline by paired t-test or Wilcoxon paired test. ****p < 0.0001; ***p < 0.001; **p < 0.01; *p < 0.05

ATPA (1 µm) application robustly increased sIPSC frequency in neonatal (5.415 ± 0.983 times, p < 0.0001; paired t-test) and juvenile (3.368 ± 0.598, p = 0.008) control mice (Fig. 2A*i, ii*, B), confirming previous results [22, 23]. Notably, ATPA effect was gradually diminishing with age in accordance with the declining expression of GluK1 towards the adulthood. In adult animals, ATPA increased sIPSC frequency, yet this effect did not reach statistical significance (2.589 ± 0.521 times, p = 0.081, paired t-test; Fig. 2A*iii*, B). In *Gad-Grik1*^−/−^ mice, ATPA had no effect on sIPSC frequency neither in neonates nor in the juvenile animals, but surprisingly, caused a slight increase in sIPSC frequency in adults (1.572 ± 0.258 times, p = 0.029, paired t-test; Fig. 2 A*i-iii*, B). These observations confirm that GluK1 activation in the hippocampal interneurons efficiently recruit GABAergic drive, particularly at earlier developmental stages.

Consistent with previous data [22, 27], ATPA application significantly decreased sEPSC frequency in neonatal (to 0.591 ± 0.063 from the baseline, p = 0.003, paired t-test) and in young (to 0.590 ± 0.069, p = 0.004) control mice, but had no effect on sEPSCs in the adult animals (Fig. 2A*i-iii*, C). The strong effects of ATPA on GABAergic transmission could result in attenuation of glutamatergic transmission indirectly, via changes in network excitability. However, ATPA application decreased sEPSC frequency also in the neonatal *Gad-Grik1*^−/−^ mice (to 0.678 ± 0.100, p < 0.006, Wilcoxon paired test), where no parallel changes in sIPSCs were observed (Fig. 2B, C). ATPA had no effect on sEPSCs in the juvenile *Gad-Grik1*^−/−^ slices, but caused a slight reduction of sEPSC frequency in the adult stage (to 0.801 ± 0.057, p = 0.030, paired t-test). These data are consistent with expression of presynaptic inhibitory GluK1 KARs in glutamatergic neurons in the neonate hippocampus [19, 22, 27], while in the juvenile mice, sEPSC frequency appears to be regulated indirectly, via the ATPA-induced changes in GABAergic drive. The cellular basis underlying the small effects of ATPA on spontaneous transmission in the adult *Gad-Grik1*^−/−^ mice remain unclear, but might represent some unspecific actions of ATPA on other KAR subtypes (e.g. [28, 29]), compensating for the loss of GluK1 in the mutants.

Inhibition of GluK1 containing KARs by ACET (200 nM) triggered increase in sEPSC frequency in both control (2.022 ± 0.344 times; p < 0.001, Wilcoxon paired test) and *Gad-Grik1*^−/−^ neonatal slices (1.503 ± 0.236 times; p = 0.031, paired t-test; Fig. 2A*iv*, D). At the same time, ACET had no effect on sIPSC frequency in either group. This further confirms that GluK1 receptors, located in glutamatergic principal neurons, tonically inhibit glutamatergic transmission during early postnatal development [19, 22]. We did not test ACET effect later on in development, since endogenous activity of GluK1 receptors is limited to early postnatal age. In line with the previous data [22] neither ATPA nor ACET application (Additional file 1: Fig. S1B, C, respectively) lead to any significant changes in the sIPSC or sEPSC amplitudes.

### Immature-type network activity in the hippocampus is regulated by GluK1 KARs expressed in both, glutamatergic and GABAergic neurons

Pharmacological manipulation of GluK1 subunit containing KARs modulate the spontaneous network activity in the neonatal hippocampus [22]. Since GluK1 receptors are expressed both in GABAergic and glutamatergic neurons [15], the cell type mainly responsible for GluK1-dependent regulation of the network excitability remains unknown.

**Fig. 3:**
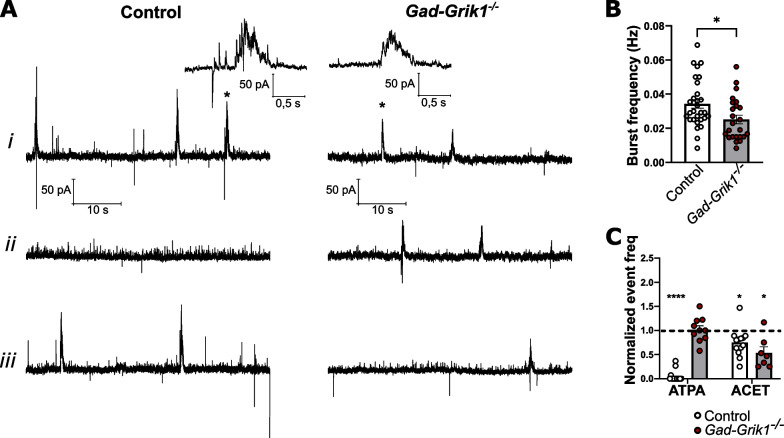
Pharmacological characterization of spontaneous network activity in neonatal *Gad-Grik1*^−/−^ mice. **A** Example traces of spontaneous activity in baseline (*i*) and upon ATPA (*ii*) or ACET (*iii*) application, recorded from CA3 pyramidal cells in acute slices from control (left) and *Gad-Grik1*^−/−^ (right) mice (P4-6). Inserts on top represent network bursts (marked with asterisk) in expanded time scale. **B** Basal frequency of spontaneous network bursts from acute control (n = 30 (21)) and *Gad-Grik1*^−/−^ (n = 24 (19)) neonatal slices (P4-6). *p < 0.05, upaired t-test test. **C** Effect of ATPA (1 µM) and ACET (200 nM) on burst frequency in control and *Gad-Grik1*^−/−^ groups. ATPA, n = 14(10) and 10(10); ACET n = 13(10) and 7(6) for control and *Gad-Grik1*^−/−^, respectively. Frequency is normalized to the baseline (dashed line). ****p < 0.0001; **p < 0.01; *p < 0.05, paired t-test

In the neonatal hippocampus, spontaneous network bursts can be readily recorded from CA3 pyramidal neurons, and consist of a slow GABAergic current, intermixed with EPSCs (Fig. 3A*i*) [22]. The frequency of these network events was significantly lower in the *Gad-Grik1*^−/−^ slices (0.025 ± 0.003 Hz), as compared to controls (0.034 ± 0.003 Hz; p = 0.016, unpaired t-test; Fig. 3A*i*, B), pointing out the important role of interneuronal GluK1 in this form of network activity. As described earlier [22], application of ATPA almost completely abolished network bursts in the control animals (to 0.050 ± 0.031 of the baseline; p < 0.0001; paired t-test), but had no effect on burst frequency in the *Gad-Grik1*^−/−^ mice (to 1.016 ± 0.081 of the baseline, p = 0.703, paired t-test; Fig. 3A*ii*, C). Interestingly, ACET application reduced the frequency of network oscillations both in slices from the control (to 0.749 ± 0.080, p = 0.018) and *Gad-Grik1*^−/−^ (0.535 ± 0.125, p = 0.011) animals (Fig. 3A*iii*, C). These pharmacological data indicate that in absence of GluK1 in the interneurons, spontaneous network bursts retain the sensitivity to GluK1 inhibition (by ACET), suggesting that ongoing regulation of network activity depends on GluK1 KARs located in principal neurons. Furthermore, loss of the ATPA dependent regulation in *Gad-Grik1*^−/−^ mice confirms that activation of GluK1 receptors in interneurons impose a strong control over the circuit excitability.

### Interneuronal GluK1 KARs regulate synchronization and propagation of activity in cultured hippocampal slices

**Fig. 4:**
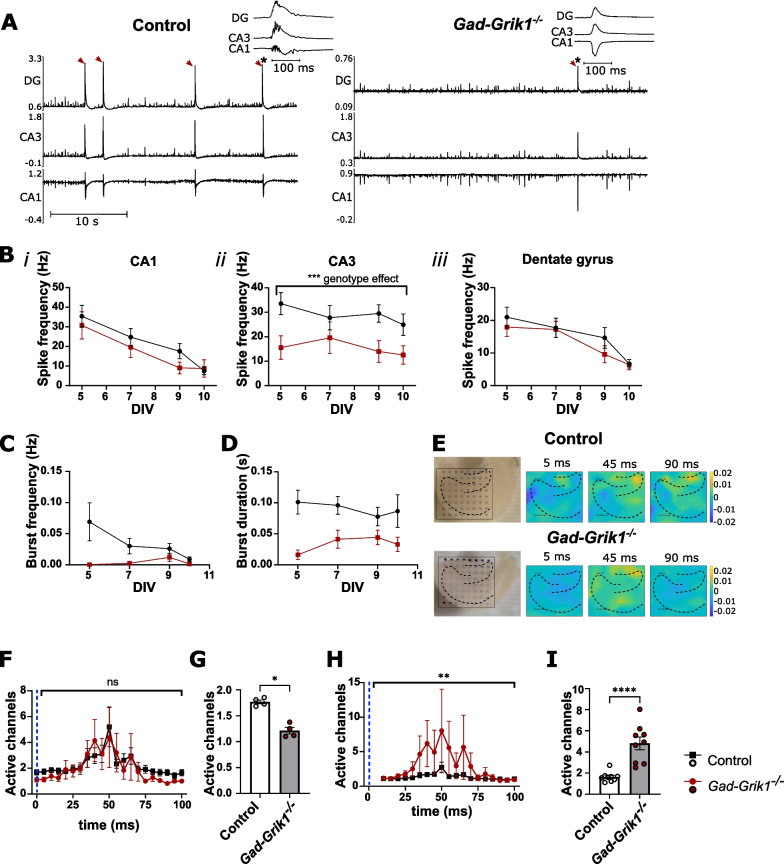
Longitudinal characterization of spontaneous network activity in organotypic hippocampal cultures from neonatal *Gad-Grik1*^−/−^ mice. **A** Example traces of the extracellular recordings across hippocampal regions in control and *Gad-Grik1*^−/−^ cultures at DIV5. The inserts show typical bursts with the CA1/CA3–DG phase shift. Red arrowheads mark network bursts. **B** Frequency of spikes detected in the CA1 (*i*), CA3 (*ii*) and DG (*iii*) regions of control (n = 12 (6)) and *Gad-Grik1*^−/−^ cultures (n = 9 (6)). Both genotypes show equal spike rates in the CA1 and DG regions, but the frequency of spikes in area CA3 is lower in the *Gad-Grik1*^−/−^ as compared to controls (p = 0.0001, 2-way ANOVA). **C** Frequency of spontaneous bursts in the CA1, CA3, DG regions of control (n = 11(6)) and *Gad-Grik1*^−/−^ slices (n = 9 (6)) at indicated DIV (p < 0.05, 2-way ANOVA). **D** Duration of spontaneous bursts in CA1, CA3, DG regions of control (n = 11(6)) and *Gad-Grik1*^−/−^ (n = 9(6)) slices at indicated DIV  (p < 0.01, 2-way ANOVA). **E** Image of the hippocampal organotypic culture on MEA probe (left) and example heat maps showing the spread of network activity following a spike in the DG, in control and *Gad-Grik1*^−/−^ cultures at 5 DIV. **F** Time course plot illustrating the mean number of spatially adjacent MEA channels (out of total 64) displaying activity during a 100 ms time period after a DG spike in control (n = 10(6)) and *Gad-Grik1*^−/−^ (n = 9(6)) cultures. The dashed blue line denotes the time point of DG spike. **G** Pooled data on the maximum number of spatially adjacent active channels during the first 20 ms following a DG spike (*p < 0.05, Mann–Whitney U-test). **H** The same data as in **F**, normalized to the first 4 time bins (20 ms) following the spike. (** p < 0.01, 2-way ANOVA). **I** Normalized number of spatially adjacent active channels 30–70 ms after the DG spike (****p < 0.0001, Mann–Whitney U-test.). Bars represent mean ± SEM. The n-values refer to number of cultures, followed by number of animals in parenthesis

Spontaneous synchronous activity is observed in the acute slices only during the first postnatal days. To further investigate the cell type-specific roles of GluK1 in network synchronization, we prepared organotypic hippocampal cultures from P4-P5 mice and recorded the spontaneous network activity longitudinally, using 8 × 8 electrode MEA probes at four time points, at DIV 5, 7, 9 and 10. In the CA1 and DG regions, the spike rates detected from *Gad-Grik1*^−/−^ mice did not differ from controls (Fig. 4A, B*i, iii*). In the CA3 region, however, slices from *Gad-Grik1*^−/−^ mice showed reduced spike rates through the whole culturing period (p = 0.0001, 2-way ANOVA; Fig. 4A, B*ii*). Furthermore, the frequency (p < 0.05, 2-way ANOVA) and duration (p < 0.01, 2-way ANOVA) of large (minimum 10 spikes within minimum 0.1 s time) bursts was significantly lower in *Gad-Grik1*^−/−^ cultures as compared to controls (Fig. 4C, D). We then further analyzed these data to look at the spread of activity in the network by computing the maximum number of spatially adjacent MEA channels displaying activity within 0.1 s time window after a spike detected in the dentate gyrus (DG). The basal excitability, assessed by the average number of clustered active channels, was lower in the *Gad-Grik1*^−/−^ slices as compared to controls (Fig. 4E–G). When the data was normalized to the first 20 ms following the spike detected in DG, the *Gad-Grik1*^−/−^ slices showed a greater increase in the number of clustered active channels (4.817 ± 0.6032 vs. 1.635 ± 0.1563 in *Gad-Grik1*^−/−^ and control, respectively, p < 0.0001, Mann–Whitney test) during the 30–70 ms period after the spike (Fig. 4H, I). Consistent with the findings from acute neonatal slices, these data from organotypic cultures indicates that ablation of GluK1 from the interneurons attenuates synchronous network activity, observed as lower frequency of the spontaneous bursting activity. At the same time, however, the spatial propagation of activity within the hippocampal network is enhanced.

### Ablation of interneuronal GluK1 leads to altered network oscillations in the adult hippocampus in vivo

**Fig. 5:**
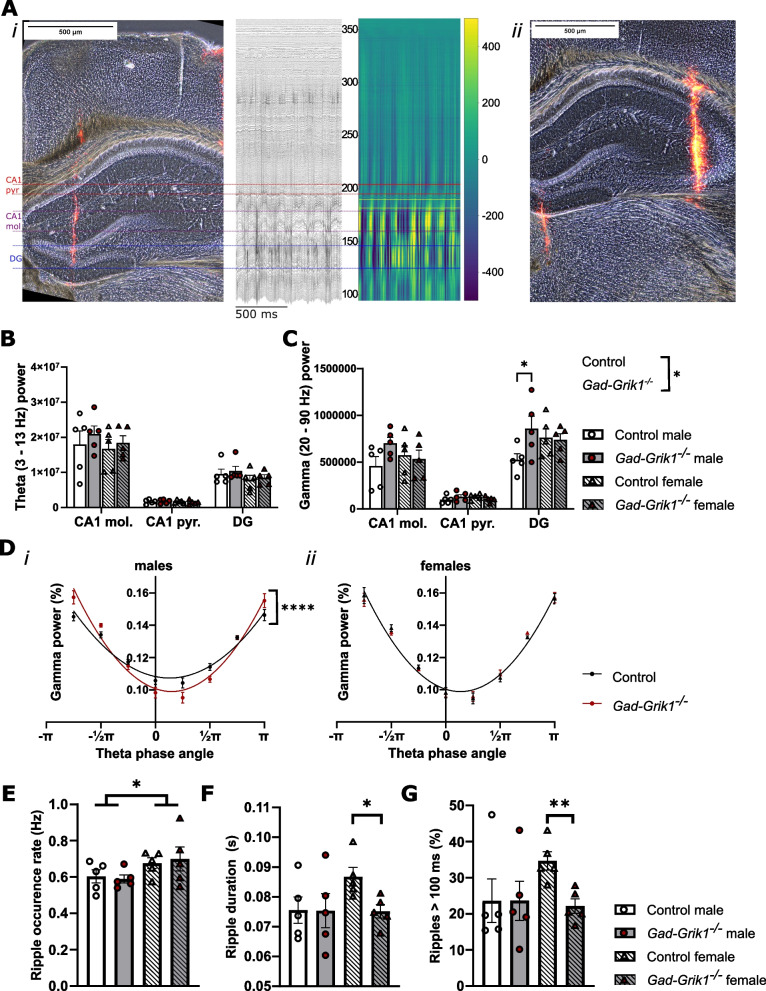
Physiological network oscillations are altered in the adult hippocampus in absence of GluK1 expression in the GABAergic neurons. **A** Image illustrating the localization of the fluorescently labelled recording electrode in a coronal section from the mouse hippocampus, in CA1 –DG (*i*) and CA3 (*ii*). The traces show an example of the LFP recording of oscillatory activity at different regions of the male control mouse hippocampus, and the heat map illustrates the color-coded voltage plots at identical time-scale after 350 Hz low pass filtering. **B** Oscillatory power in the theta (4–12 Hz) frequency range for channels located in the CA1 *stratum moleculare* (CA1 mol), CA1 *stratum pyramidale* (CA1 pyr) and dentate gyrus (DG), for male and female control and *Gad-Grik1*^−/−^ mice (n = 5 / group). **C** Oscillatory power in the gamma (20–90 Hz) frequency range, for the same recordings as in **B**. * p < 0.05, 2-way ANOVA, Tukey’s correction for multiple comparisons). **D** Oscillatory power in the gamma range, as function of the theta phase angle divided into 8 equal sized bins. The solid lines represent second order polynomial (quadratic) curve fit. Gamma power in the CA1 is more strongly modulated by theta phase (**** p < 0.0001, n = 5, quadratic regression, sum-of-squares F test) in male (*i*), but not in female (*ii*) (n = 5, quadratic regression, sum-of-squares F test) *Gad-Grik1*^−/−^ mice. **E** Rate of occurrence of ripple oscillations in the CA1 pyramidal layer. * p < 0.05, 2-way ANOVA. **F** Duration of ripple oscillations detected in the CA1 pyramidal layer. *p < 0.05, unpaired t-test. **G** Percentage of ripples lasting more than 100 ms. All data from idle or resting epochs, detected from videos recorded simultaneously with the electrophysiological recording. ** p < 0.005, unpaired t-test. Bars in all panels represent mean ± SEM

Interneurons regulate all the main types of rhythms occurring in the awake hippocampus: the theta (4–12 Hz), gamma (20–150 Hz) and sharp wave ripple (SWR) oscillations. In order to investigate the role of interneuronal GluK1 in physiological oscillations in the adult hippocampus, we performed a series of recordings in awake, head-fixed mice, using multichannel linear probes spanning over the CA1 and DG regions and separately, the CA3 region of the hippocampus (Fig. 5A*i* and *ii,* respectively). Since the oscillatory power in the hippocampus depends on locomotion and exploratory activity [30, 31], the recorded signals were divided into epochs of idle and running based on video analysis of the mouse activity.

The *Gad-Grik1*^−/−^ mice did not differ from their littermate controls in oscillatory power in the theta frequency range (Fig. 5B) either during the resting state, nor while they were actively moving (Additional file 2: Fig. S2), with the exception of the high frequency (7–13 Hz) sub-band of theta power being higher in the *Gad-Grik1*^*−/−*^ male mice than controls when the mice were moving (Additional file 2: Fig. S2D*i*, p = 0.0167, Holm-Šídák). However, oscillatory power in the gamma range was elevated in the *Gad-Grik1*^−/−^ males, in the DG (p < 0.05, 2-way ANOVA, Tukey’s post hoc test) independent on the behavioral state (Fig. 5C). On the contrary, in the female mice (n = 5) there were no differences between genotypes in any of the frequency ranges we investigated (Fig. 5B, C).

As theta-gamma coupling occurs in the hippocampus during running and REM-sleep in rodents [30, 32] and correlates with learning of spatial tasks [33–35], we next looked at how gamma power is modulated by theta phase in two genotypes. In the *Gad-Grik1*^*−/−*^ male mice the gamma power in CA1 region was more strongly modulated by theta phase than in the controls (Fig. 5D*i*, p < 0.0001, quadratic regression, sum-of-squares F test). In females there were no differences between genotypes in the theta phase to gamma power-cross frequency modulation (Fig. 5D*ii*).

SWRs consists of the sharp wave detected as large amplitude negative deflection in the LFP in CA1 *stratum radiatum*, followed by a high frequency (150–200 Hz) ripple oscillation in the CA1 pyramidal layer. We used the Kay ripple detection method [36] to identify ripple oscillations in the 150–200 Hz frequency range in the CA1 pyramidal layer. Ripples were detected from epochs where the mouse was idle or resting, and the occurrence rate of ripples was calculated across the full duration of such epochs. Ripples occurred as frequently in both genotypes, and were more frequent in females than in males (2-way ANOVA gender effect p = 0.0393, Fig. 5E). However, in female mice the duration of ripples was shorter (p = 0.0180, two-tailed unpaired t-test, Fig. 5F) and the percentage of ripples exceeding 100 ms in duration was lower (p = 0.0047, two-tailed unpaired t-test, Fig. 5G) in *Gad-Grik1*^−/−^ mice as compared to controls.

### Ablation of interneuronal GluK1 leads to reduced general activity, novel object avoidance and changes in cognitive flexibility

**Fig. 6:**
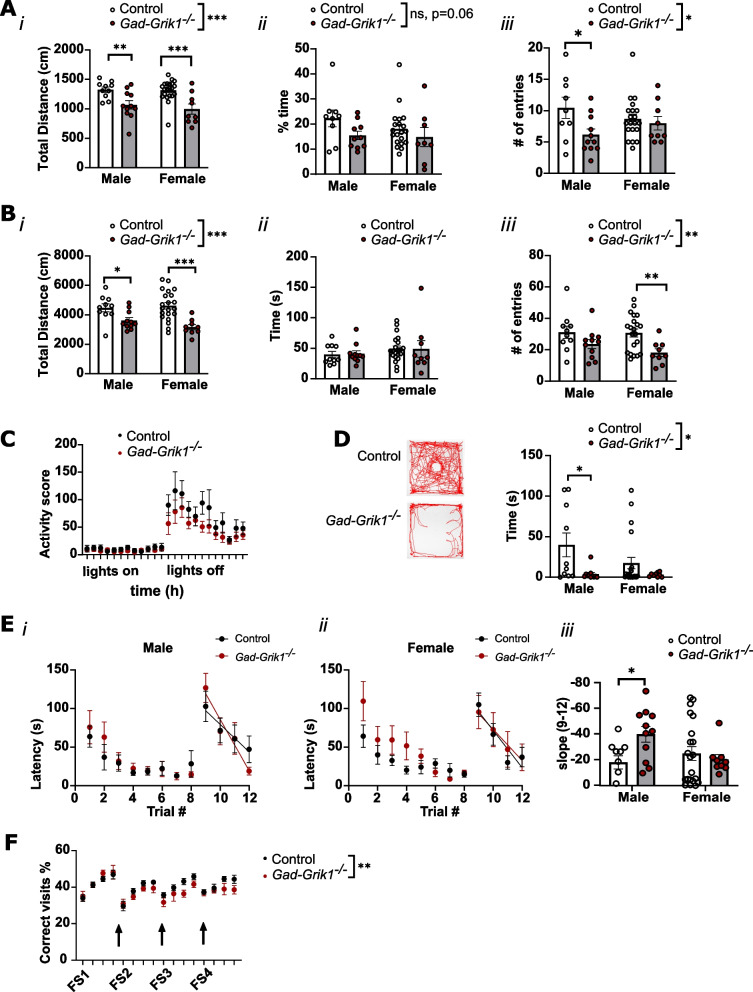
Behavioral phenotype in *Gad-Grik1*^−/−^ shows reduced activity and altered cognitive flexibility. **A** EPM test. (*i*) total distance traveled (*ii*) time spent in the open arms (*iii*) entries to the open arms. * p < 0.05; ** p < 0.01; *** p < 0.001; Holm-Šídák posthoc test after 2-way ANOVA. **B** OF test. (*i*) total distance traveled (*ii*) time spent in the central zone (*iii*) entries to the central zone. * p < 0.05; ** p < 0.01; *** p < 0.001; Holm-Šídák posthoc test after 2-way ANOVA. **C** Home-cage activity for single-housed male control and *Gad-Grik1*^−/−^ mice. The activity score is plotted against time of the day, with 1 h bins. *** p < 0.001, 2-way ANOVA. **D** OF test, with a novel object placed in the center of the field. Example tracks traveled by a control and *Gad-Grik1*^−/−^ mouse, and pooled data for the time spent in the central zone. * p < 0.05; Holm-Šídák posthoc test after 2-way ANOVA. **E** Barnes maze test. Latency to the correct hole in the Barnes maze during training (trials 1–8) and after relocation of the escape box (trials 9–12) for male (*i*) and female (*ii*) control and *Gad-Grik1*^−/−^ mice. (*iii*) Pooled data on the slope of the learning curve between trials 9–12 (* p < 0.05, two-tailed unpaired t-test). **F** Flexible sequencing task in the IntelliCage. Arrows denote location reversal times. *Gad-Grik1*^−/−^ female mice show less correct visits and more incorrect visits after the second reversal. ** p < 0.01, 2-way ANOVA. For all the behavioral data, controls, n = 10 and n = 21, *Gad-Grik1*^−/−^ n = 11 and n = 9, for males and females, respectively. As an exception, control, n = 15, *Gad-Grik1*^−/−^ n = 9 for the data in panel **F**

The differences we found in excitability and synchronous network activity both in vitro and in vivo led us to investigate how knocking out interneuronal GluK1 affects the behavior of mice in adulthood. For this purpose, we performed a battery of behavioral tests on littermate control and *Gad-Grik1*^−/−^ mice. First, we looked at the activity and possible anxiety-like phenotypic features using the open field (OF) and elevated plus maze (EPM) tests. The total distance traveled by *Gad-Grik1*^−/−^ mice during both the EPM (Fig. 6A*i*) and OF tests (6B*i*) was significantly lower as compared to controls in both males and females, indicating lower level of activity (p < 0.0001 for both tests, 2-way ANOVA). This phenotype was confirmed by automated monitoring home-cage activity in single-housed male control and *Gad-Grik1*^−/−^ mice over 6 days. *Gad-Grik1*^−/−^ mutants were less active as compared to their littermate controls, particularly during the dark (active) phase (full time period p = 0.0003, dark period p = 0.0008, light period p = 0.08; 2-way ANOVA; Fig. 6C).

Although GluK1 containing KARs, located in amygdala interneurons have been implicated in anxiety-like behaviors in rodents (e.g. [37, 38]), only a minor features of anxiety were observed in the present tests. There were no significant differences in the time spent in the center zone of the OF arena (6B*ii*), yet the number of entries to the central zone was significantly lower in the *Gad-Grik1*^−/−^ mice as compared to controls (p = 0.0026, 2-way ANOVA; Fig. 6B*iii*). In the EPM, the percentage of time spent in the open arms was slightly but not significantly different between genotypes (p = 0.059, 2-way ANOVA; Fig. 6A*ii*), and the number of entries to the open arm was significantly lower in the *Gad-Grik1*^−/−^ mice as compared to controls (p = 0.0285, 2-way ANOVA; Fig. 6A*iii*). Interestingly, when a novel object was placed in the middle of the center zone in the OF test, *Gad-Grik1*^−/−^ spent significantly less time in the object zone as compared to controls (p = 0.0057, 2-way ANOVA; Fig. 6D). This phenotype was apparent in both male and female mice, but in post-hoc testing reached significance only in males (p = 0.0149, Holm-Šídák).

Since spontaneous network oscillations, especially theta-nested gamma oscillations are associated with spatial learning, memory functions and cognitive flexibility [30, 34, 35], we next looked at the performance of the *Gad-Grik1*^−/−^ mice in the Barnes maze (BM) spatial learning and memory task. The task consisted of 2 parts: (1) training trials 1–8, where the animals learned to find the target location, i.e. a hole leading to the escape box and (2) training trials 9–12 which took place after the escape box had been relocated (paradigm shift). We ignored the first three training trials, as they are highly affected by the environmental novelty the animals face. In the training trials 4–8 we did not see differences between the genotypes in either gender (Fig. 6E), indicating that the mutant mice were able to learn the spatial location of the escape box equally well as the controls. After the relocation of the escape box (training trials 9–12) the male *Gad-Grik1*^−/−^mice showed a steeper slope in relearning the correct target location (Fig. 6E*i, iii*) as compared to littermate controls (p < 0.05, two-tailed unpaired t-test). In females, there were no differences in the slope of relearning the BM task (Fig. 6E*ii*, *iii*).

To further assess the cognitive flexibility of the *Gad-Grik1*^−/−^ mice, we used the flexible sequencing task in the IntelliCage [39]. In this test, female mice are group housed in a home cage with automated tracking and remotely controlled location of a reward. Following habituation to the novel environment, the animal is trained to obtain a reward by making a nose poke. In the learning test, the mouse obtains the reward by shuttling between two diagonally opposite corners of the cage, after which the pattern of reward locations is changed for reversal learning. We found that control and *Gad-Grik1*^−/−^ females were able to learn the task equally well. However, after the first two reversals, the *Gad-Grik1*^−/−^ females started showing less correct visits as compared to the controls. Statistical testing indicated a significant genotype effect over the whole test (p = 0.0030, 2-way ANOVA) and in reversals 3 and 4 (p = 0.0021 and p = 0.0299, 2-way ANOVA), but not reversals 1 and 2, individually (Fig. 6F). These data are consistent with alterations in cognitive flexibility, also in females.

## Discussion

KARs have established functions in modulation of synaptic transmission, plasticity and neuronal excitability, both during development and in the adult brain [12, 14, 40]. However, how the various modulatory functions attributed to different types of KARs, expressed in distinct cell types and subcellular compartments, control the physiological activity at the level of neuronal networks is less well understood. To start to investigate this question, we generated a mouse model lacking GluK1 subunit containing KARs specifically in the GABAergic neurons. Electrophysiological characterization of these mice indicate a critical role for GluK1 KARs, expressed in GABAergic interneurons, in maturation of GABAergic transmission and network synchronization during early postnatal development. Furthermore, consistent with prolonged interneuronal dysfunction, aberrant oscillatory activity in the gamma frequency band as well as mild alterations in behavioral tasks requiring cognitive flexibility were detected in the adults.

### Cell-type specific mechanisms involved in regulation of neonatal network activity by GluK1

The physiological functions of GluK1 subunit containing KARs have been comprehensively characterized in the developing hippocampus, by using GluK1 selective pharmacological tools [19, 22, 23, 41, 42]. These data suggest that activation level of GluK1 KARs in the neonatal hippocampus is finely tuned to permit the typical rhythmic activity of the immature circuitry [11], yet the KAR subpopulations involved in this regulation are not known.

The frequency of spontaneous network bursts was significantly reduced in *Gad-Grik1*^−/−^ mice, indicating a critical role of interneuronal GluK1 receptors in regulation of network synchronization. This phenotype as well as the parallel decrease in spontaneous GABAergic synaptic transmission in *Gad-Grik1*^−/−^ mice could be fully explained by lower firing rate of interneurons, due to loss of GluK1-dependent regulation of SK potassium channels [42]. Decrease in the mean firing rate of GABAergic neurons is sufficient to elevate the threshold for generation of synchronous bursts in the immature network, shown specifically for somatostatin-expressing interneurons [43]. GluK1 coupling to SK-channels is developmentally downregulated and accordingly, there was no difference in sIPSC frequency between *Gad-Grik1*^−/−^ and control mice during adolescence and adulthood. However, we cannot rule out the possibility that various homeostatic compensatory mechanisms that can efficiently balance excitability of immature neuronal networks contribute to the loss of differences between the genotypes during maturation of the circuitry.

MEA recordings from cultured *Gad-Grik1*^−/−^ slices indicated that in addition to lower occurrence of network bursts, activity generated in the DG spread more efficiently across the hippocampus in the absence of interneuronal GluK1. Previously, it has been shown that augmented inhibition in response to pharmacological activation of GluK1 receptors restricts propagation of epileptiform activity from one hemisphere to other [44]. Our results extend these findings to more physiological context and suggest that activation of interneuronal GluK1 by endogenous glutamate limits the size of activated neuronal population by enhancing GABAergic inhibition. Thus, in the immature hippocampus, interneuronal GluK1 receptors promote local synchrony, but restrict spatial propagation of the activity.

Pharmacological characterization of the spontaneous activity in *Gad-Grik1*^−/−^ mice was performed to further understand the cell-type specific mechanisms involved. Activation of GluK1 receptors by ATPA results in robust increase in sIPSC frequency, which has been attributed to activation of somatodendritic GluK1-containing KARs in GABAergic neurons [22, 23]. This effect was completely abolished in the *Gad-Grik1*^−/−^ mice, thus confirming the previous conclusion and also validating the mouse model. ATPA application also results in depression of glutamatergic transmission at the immature synapses [22, 27], via activation of inhibitory presynaptic GluK1 receptors [27]. This effect persisted in neonatal but not juvenile *Gad-Grik1*^−/−^ mice, confirming that GluK1 receptors are expressed in glutamatergic terminals in the hippocampus during the first postnatal week.

Blocking GluK1 KARs attenuated network bursts in both control and *Gad-Grik1*^−/−^ mice, indicating that endogenously active GluK1 receptors in principal neurons also contribute to synchronization of the neonatal network. In the adults, alterations in glutamatergic drive to interneurons modulate network oscillations [45–47]. Similarly, ongoing GluK1 dependent regulation of glutamate release to interneurons [22] may promote generation of synchronous network bursts in the neonatal hippocampus.

Together, the present data confirm that network activity in the neonatal hippocampus is regulated by distinct subpopulations of GluK1-containing KARs, located in GABAergic interneurons and glutamatergic principal cells. GluK1 expressed in GABAergic neurons facilitates local synchronization of immature hippocampal network, presumably via regulation of interneuron excitability and firing rate. In parallel, endogenous activity of GluK1 KARs expressed in principal neurons maintains glutamatergic drive to interneurons and facilitates network bursts.

### Loss of GluK1 in GABAergic interneurons associates with small changes in hippocampal network dynamics and behavioral flexibility in adults

Pharmacological studies have implicated GluK1 KARs in modulation of hippocampal theta oscillations in vivo [48] and gamma oscillations in vitro [49–51], yet no previous data on rhythmic network activity in mice lacking GluK1 exists. Our recordings in awake mice indicated that ablation of GluK1 in GABAergic interneurons increases oscillatory power within the gamma frequency band in males. In addition, the theta-modulation of gamma oscillations was stronger in *Gad-Grik1*^−/−^ males as compared to controls. Although interneuronal GluK1 has been previously implicated in regulation of synaptic and network activity in the theta frequency [48, 52, 53], no significant differences in theta activity were detected in *Gad-Grik1*^−/−^ mice. Interneuronal KARs are composed heteromeric combinations of GluK2 and GluK1 subunits [28, 29, 54], which can functionally compensate for each other [54] and possibly explain the lack of some phenotypes in the adult *Gad-Grik1*^−/−^ mice. Alternatively, theta activity involves GluK1 receptors that are expressed in other cell types than GABAergic interneurons.

Gamma oscillations are modulated by glutamate receptors in interneurons [46, 47, 55]. In particular, ablation of NMDA receptors in parvalbumin (PV) positive interneurons augments gamma oscillation power [46, 55]. GluK1 KARs modulate the excitability of PV neurons in the adult brain [37] providing a putative mechanism for the altered gamma activity in the *Gad-Grik1*^−/−^ male mice. However, since previous work detected no effects of GluK1 antagonism on gamma oscillations in the hippocampus [48], it is also possible that the delayed maturation of the GABAergic activity in the *Gad-Grik1*^−/−^ mice contributes to the phenotype, for example by causing long-lasting structural changes in the circuitry underlying gamma frequency synchronization. Interestingly, no differences between genotypes were detected in the females. Instead, females had a distinctive phenotype in sharp wave ripple oscillations, which were shorter in length in *Gad-Grik1*^−/−^ females. The hippocampal SWRs are driven by strong excitatory drive from the CA3, combined with synchronous local activation of interneurons in the CA1 region [56, 57]. Therefore, both the impaired gamma and high-frequency ripple oscillations could be explained by lower excitability of GluK1 deficient interneurons, which reduces synchronization in the neonate as well as in the adult circuitry [43, 47]. Why *Gad-Grik1*^*−/−*^ genotype results in different phenotype in terms of oscillatory activity in males and females is currently not clear. Interestingly, we could observe, slight, but not significant gender differences in control animals with elevated gamma power in females, as previously described [58, 59], and shorter SWRs in males. To our knowledge, even though SWRs are associated with features known to have gender specificity (as e.g. social memory [60], hypothalamic circuits [61], glucose metabolism [62]) there are no reports on SWRs gender differences in rodents. In humans, however, sleep spindles (associated with SWRs [63]) are shorter, less frequent and have lower power in boys [64].

Theta and gamma oscillations as well as the phase relationships between them are considered critical for memory encoding [65, 66]. Despite the changes observed in gamma power and theta-gamma cross frequency coupling, *Gad-Grik1*^−/−^ mice performed equally well as their littermate controls in a spatial learning task. However, *Gad-Grik1*^−/−^ males relearned the new location of the escape box in the Barnes maze slightly faster as compared to controls, suggesting improved reversal learning and cognitive flexibility. In contrast, females showed a mild defect in cognitive flexibility in a task that requires the ability to relearn the correct spatial sequence several times over the course of several days. Interestingly, suppression or disruption of ripple oscillations has been shown to impair spatial learning and memory [67, 68] while prolongation of ripple oscillations through optogenetic stimulation improved performance in a spatial memory task [69].

While mice lacking GluK1 display changes in drug-induced behavioral plasticity [70–72] and anxiety-like behaviors [37, 38], a role for GluK1 in reversal learning and cognitive flexibility has not been reported previously. Interestingly, mice lacking GluK2, the KAR subunit highly expressed in GABAergic interneurons but also in many other cell types [54, 73], display reduced locomotor activity, faster spatial learning and impaired spatial reversal learning [74] phenotypes similar to those observed in *Gad-Grik1*^*−/−*^. Since GluK1 and GluK2 form functional heteromeric receptors in GABAergic interneurons [28, 29, 54], it is possible that the shared behavioral phenotypes in GluK2^−/−^ mice and *Gad-Grik1*^−/−^ depend on interneuronal KARs.

In the present study, we found only mild increase in anxiety-like behaviors in the standard open field and EPM tests, suggesting that GluK1 expression in GABAergic neurons is only partially responsible for this phenotype. Interestingly, however, *Gad-Grik1*^−/−^ mice showed strong avoidance of a novel object placed in the center of the open field. The novel object induces an approach-avoidance conflict, where avoidance is interpreted as an indication of anxiety [e.g. [75]]. On the other hand, the robust avoidance may also reflect fear of novelty, rather than lack of incentive motivation to explore novel stimuli. Both interpretations are consistent with previous findings suggesting a role for interneuronal KARs in fear and anxiety—like behaviors [37, 38, 72].

## Materials and methods

### Animals

Experiments were performed using the following mouse lines: *Grik1*^*tm1c*/*tm1c*^and *Gad2-Grik1*^*tm1d/tm1d*^. *Grik1*^*tm1a*^ (KOMP)Mbp mice in C57BL/6N background were obtained from KOMP repository (UC Davis) and crossed with CAG-Flp transgenic line to produce a floxed conditional knock-out mice (*Grik1*^*tm1*c/tm1c^) [20]. The *Grik1*^*tmc1/tm1c*^ mice were crossed with *Gad2*^*tm2(cre)Zjh*^/J mice (expressing Cre under the *Gad2* promoter) to obtain the *Gad2-Grik1*^*tm1d/tm1d*^ line (referred to as *Gad-Grik1*^−/−^). Homozygous animals were used for all the in vitro experiments. *Grik1*^*tm1c/tm1c*^ mice heterozygous for the *Gad2-Cre*, with littermate *Grik1*^*tm1c*/*tm1c*^ controls were used for the behavioral and in vivo experiments. All the experiments were done in accordance with the University of Helsinki Animal Welfare Guidelines and approved by the Animal Experiment Board in Finland.

### In vitro electrophysiology

*Preparation of acute slices.* Acute sagittal sections (300–400 μm) were prepared from brains of neonatal (P4–P6) male or female mice, juvenile (P18–P21) or adult (> P50) male mice using standard methods. Briefly, mice were decapitated under isoflurane anesthesia, the brains were extracted and immediately placed in carbogenated (95% O_2_/5% CO_2_) ice-cold sucrose-based dissection solution containing (in mM): 87 NaCl, 3 KCl, 7 MgCl_2_, 1.25 NaH_2_PO_4_, 0.5 CaCl_2_, 50 sucrose, 25 glucose, 25 NaHCO_3_ (in majority of the experiments) or in low Ca^2+^–high Mg^2+^ dissection solution, containing (in mM): 124 NaCl, 3 KCl, 1.25 NaH_2_PO_4_, 26 NaHCO_3_, 15 glucose, 10 MgSO_4_, 1 CaCl_2_ (part of experiments in the neonatal mice). The hemispheres were separated and slices were cut using a vibratome (Leica VT 1200S). Slices containing the hippocampus were placed into a slice holder and incubated for 30 min in carbogenated, warm (34 °C) High-Mg^2+^ ACSF (in mM): 124 NaCl, 3 KCl, 1.25 NaH_2_PO_4_, 26 NaHCO_3_, 15 glucose, 3 MgSO_4_*7H_2_O, 2 CaCl_2_. Slices were then maintained at room temperature.

*Electrophysiological recordings from acute slices.* After 1–5 h of recovery, the slices were placed in a submerged heated (32–34 °C) recording chamber and perfused with standard ACSF, containing (in mM): 124 NaCl, 3 KCl, 1.25 NaH_2_PO_4_, 26 NaHCO_3_, 15 glucose, 1 MgSO_4_, 2 CaCl_2_ (95% O_2_ / 5% CO_2_) at the speed of 1–2 ml/min. Whole-cell patch-clamp recordings were done from CA3 principal neurons under visual guidance using patch electrodes with resistance of 2–7  MΩ, filled by low-chloride filling solution, containing (in mM): 135 K-gluconate, 10 HEPES, 2 KCl, 2 Ca(OH)_2_, 5 EGTA, 4 Mg-ATP, and 0.5 Na-GTP, 280–285 mOsm (pH 7.2–7.35. Multiclamp 700B amplifier (Molecular Devices), Digidata 1322 (Molecular Devices) or NI USB-6341 A/D board (National Instruments) and WinLTP version 2.20 or pClamp 11.0 software were used for data collection, with low pass filter (6 kHz) and sampling rate of 5 or 20 kHz. In all voltage-clamp recordings, uncompensated series resistance (Rs) was monitored by measuring the peak amplitude of the fast whole-cell capacitance current in response to a 5 mV step. Only experiments where Rs < 30 MΩ, and with < 20% change in Rs during the experiment, were included in analysis. The drugs were purchased from Tocris Bioscience (ACET: 2728, ATPA: 1107).

*Preparation of organotypic hippocampal cultures on MEA probes.* Before use, MEA probes (MED-P515A, Alpha MED Scientific, 8 × 8 electrodes; electrode size 50 × 50 μm; array size 1 × 1 mm; spacing 150 μm) were sterilized with 70% ethanol for one hour, washed with sterile water and dried under UV-light for one hour. For adhesive coating, the probes were treated with sterilized poly-L-lysine (Sigma-Aldrich) diluted 1:10 with MQ-water (1 ml dilution/probe) over night at RT. Next day, before starting culturing, the probes were washed 3 times with sterile MQ-water.

P4-P5 mice were quickly decapitated, the whole head was briefly immersed in 70% EtOH and transferred into a laminar hood in Gey′s Balanced Salt Solution (GBSS) (G9779, Sigma). The brain was extracted, the hemispheres were separated and placed on a stage, and covered with 1–2 ml of low melting point agarose gel. 350 µm coronal slices were cut using a tissue chopper (McIlwain), the slices were placed in cold GBSS and the hippocampus was extracted from the slices. The hippocampi were placed on Poly-L-lysine coated MEA probes with 280 µl preheated Neurobasal A (Gibco) medium, supplemented with 2% B27-supplement (Gibco), 2 µM L-glutamine and chloramphenicol (NB-A medium). The medium was changed and the probes were put in a petri dish containing 1–2 ml sterile H_2_0, and placed in the humidified cell culture incubator (+ 37 °C, CO_2_ 5%), first 60 min without rocking and then on a slowly moving plate rocker. The medium was added or changed in the cultures according to the following plan: Days in Vitro (DIV) 0: 280 µl NB-A, DIV 1: add 20–60 µl NB-A, DIV 2: change ~ 70% NB-A, 3 DIV 2: add 20–60 µl NB-A, DIV 4: change ~ 50% BrainPhys (BrainPhysTM Neuronal medium supplemented with SM1 neuronal supplement (Stemcell Technologies), and 0.5 mM L-glutamine), followed by adding 20–60 µl BrainPhys or changing ~ 70% BrainPhys on alternating days until 10 DIV.

*Electrophysiological recordings from cultured organotypic hippocampus slices.* The spontaneous activity of the slices was recorded at four time points, at DIV5, 7, 9 and 10. Before recording, on 5, 7, and 9 DIV 20–60 µl fresh medium was added and 10 DIV 70% medium was changed, and the cultures were left for minimum one hour in the incubator before starting the recording. The slices on the probes were then imaged using Leica MZ10F microscope (Leica Microsystems GmbH, Wetzlar, Germany) and Qimaging Rolera Bolt Scientific camera (Teledyne QImaging, BC, Canada) to ensure healthy morphology and to confirm electrode positions. Slices that had detached from the probe or had clear, visually detectable damage such as holes in the tissue were rejected. The probes were connected to the MED64 system by the MED-C03 connector inside the recording incubator (+ 37 °C, CO_2_ 5%). The signal was monitored for 30 min for stabilization, followed by 10–30 min of recording using the MED64-amplifier (MEDA64HE1) and Mobius software (Alpha MED Scientific), with 20 kHz sampling rate, 0.1 Hz high pass and 5 kHz low pass filtering and 5 mV voltage range.

### In vivo electrophysiology

*Head plate implantation surgery.* The mice were anesthetized using ≥ 4% isoflurane. Carprofen (Rimadyl vet, 5 mg/kg *s.c*.), dexamethasone (2 mg/kg *s.c*.) and buprenorphine (Bupaq vet, 0.05 mg/kg *s.c*.) were injected to reduce pain, inflammatory response and brain edema. The mice were then placed on a 37 °C heating pad in the stereotaxic device. The depth of anesthesia was adjusted to 1.5–2% isoflurane and both eyes were covered with ophthalmic ointment (Viscotears, Novartis) to protect the cornea from dehydration. The hair on top of the head was trimmed and the skin was disinfected by povidone-iodine (Betadine). Lidocaine (0.5%, max 5 mg/kg) was injected under the scalp for local anesthesia, and scalp and periosteum were removed. The surface of the skull was roughened using a large diameter drill bit and carefully cleaned. Tissue adhesive (3 M Vetbond) was used to seal the edges of the wound. The locations for craniotomies were identified and marked using a drill. The lightweight stainless-steel head plate with round 8.2 mm opening (Neurotar model 5) was attached to the skull using a small amount of super glue (Loctite precision), and the sides of the head plate were covered with dental cement (3 M RelyX). After operation the animals were allowed to recover on the heating pad, and placed in the home cage with some water-soaked soft food pellets. The weight of the mice was monitored and carprofen (Rimadyl vet, 5 mg/kg *s.c.*), and buprenorphine (Bupaq vet, 0.05 mg/kg *s.c.*) were injected for two days after the surgery for post-operative care. In case of weight loss, 0.1–0.3 ml glucose (20 mM; max: 20 ml/kg) solution in saline was *i.p*. injected in the first 3 days after operation.

*Habituation, craniotomy and electrophysiological recording from awake, head-fixed mice.* After recovery from the head-plate surgery (at least 3 days), the mice were habituated to being head-fixed for 5 min, 10 min, 15 min, 30 min and 1 h on consecutive days before the recordings. On the day of recording or one day before, a small craniotomy was made for placing the recording probe. Briefly, the mice were anesthetized using ≥ 4% isoflurane. Carprofen (Rimadyl vet, 5 mg/kg *s.c*.) was injected, and the mice were placed on a 37 °C heating pad in the stereotaxic device. The depth of anesthesia was adjusted to 1.5–2% isoflurane and both eyes were covered with ophthalmic ointment (Viscotears, Novartis). A grounding screw for connecting the ground electrode was attached to the skull using dental cement (3 M RelyX). A small circular craniotomy was drilled in the right hemisphere on top of the recording site (RC: (− 2)–(− 2.1) mm, ML: 0.9–1.3 mm for CA1 and DG; RC: (− 2.1)–(− 2.3) mm, ML: 2.3–2.6 mm for CA3) and covered with silicone adhesive (Kwik-Sil, World Precision Instruments). A bath from Kwik-Sil with ca 10 mm high walls was constructed on top of the head plate opening. The mice were let to recover in the home cage for at least one hour prior the recording.

For recording, the mice were head-fixed on top of a rotating ball. The ground electrode was attached to the grounding screw and a linear probe (Neuropixels 1.0) was placed on the surface of the brain and inserted horizontally into the desired depth (3.5 mm for CA1 and DG, 3.7–4.0 mm for CA3). Before insertion the probe was stained by the DiI (V22885, TermoFisher) droplet, to allow post-experimental histological evaluation. The bath on top of the head plate was filled with sterile filtered saline (0.9% NaCl). After 15 min stabilization period, simultaneous recordings of local field potential (LFP) and multi-unit activity (MUA) were performed at 30 kHz sampling rate (2500 Hz online down sampling for the LFP signal) using the IMEC Neuropixels acquisition system and Spike GLX software. A custom made Python application was used for video acquisition. After the recording, the craniotomy was covered with Kwik-Sil. The recordings for CA1/DG and CA3 were done on consecutive days. Within one week from recording the mice were transcardially perfused by PBS followed by 4% PFA and the brains were collected for histological analysis.

### Data analysis

#### Analysis of the in vitro electrophysiological recordings

The frequency and amplitude spontaneous IPSCs (sIPSCs), EPSCs (sEPSCs) and network bursts were analyzed using Minianalysis 6.0.0.3 (Synaptosoft). For the sIPSCs (outward current) and sEPSCs (inward current), the amplitude threshold was set two times the baseline RMS noise level. Detected events were verified manually. Network bursts were identified on the base of slow outward current, with an amplitude and duration of at least 10 pA and 100 ms, respectively. For sIPCSs and sEPSCs, at least 5 min per condition or 200 events were analyzed, and the network bursts were analyzed from the complete recording. Cells with spontaneous event frequency less than 0.008 Hz (0.5 event/min) were excluded.

For the MEA data analysis, representative channels were selected from the CA1, CA3 and DG regions based on the images taken before data acquisition. Spikes and bursts (minimum number of spikes = 10, minimum duration of burst = 0.1 s) from the MEA recordings were detected using NeuroExplorer 5.205 functions DetectSpikes (using default parameters) and Bursts (using the Surprise algorithm with default parameters). Dentate spikes for MEA activity spread analysis were detected using Spike2 (v. 9.03a Cambridge Electronic Design), and cluster activity following the DG spikes was detected using a custom made Matlab script. Before a calculation of activity clusters traces were downsampled (5 kHz) and demeaned (1 s window with 50% overlap). We defined an activity cluster as a spatially adjacent (bwconncomp MATLAB function) group of channels in which local field potential is higher (for positive clusters) or lower (for negative clusters) than a threshold value (2 and − 2 standard deviation of the signal, respectively). Activity clusters were calculated in 21 time points near each spike (100 ms window centered at the spike timestamp with 5 ms time lag). If there were several clusters at one time point, only a cluster with a maximum size was used for further calculations.

### Detection of epochs of rest and movement

Deep Lab Cut 2.2.0.2 [76, 77] was used to track 7 points (the front paws, the mouth, and the tip, philtrum and nares of the snout) in the videos recorded during the in vivo data acquisition. We used ResNet-50-based neural network [78, 79] to train the model for detection and tracking of the mouse body parts. The DLC tracking data was passed to SimBA 1.3.12 [80] to detect epochs of movement and rest. The SpikeGLX command line tool CatGT (v. 2.4) was used to extract event times from the video TTL signal that was recorded to synchronize video frames to the electrophysiological signals. A custom-made Python program was used to extract the electrophysiological data corresponding to each detected epoch.

### Analysis of oscillatory power and cross-frequency coupling

The data was processed using custom-made Python programs, with MNE (v. 0.24.0), Numpy (v. 1.19.5), ripple_detection (v.1.2.0) modules and readSGLX from the SpikeGLX_Datafile_Tools package. The signals were low-pass filtered to 350 Hz and DC-components were removed by subtracting channel means. For each epoch, a time–voltage–plot covering all channels was plotted, and epochs with artifacts or noise were manually discarded based on the plotted figures.

Channels for analysis were selected on the basis of post-mortem histological investigation (i.e. recording sites of fluorescently-marked electrodes), LFP signal properties (i.e. amplitude, phase shift, CA1 ripples) and visually identified spike patterns. 10–20 channels representing (i) the CA1 *stratum pyramidale*, CA1 *stratum moleculare* and Dentate Gyrus (DG) regions and (ii) the CA3 *stratum pyramidale* and CA3 *stratum moleculare* were selected for analysis. The recorded signals in selected channels were divided into 3 s epochs of idle and running based on video analysis of the mouse activity.

For each sub region separately, complex Morlet wavelet convolution was performed using the MNE tfr_array_morlet-function, separately for theta (3–12 Hz) and gamma (20–90 Hz) frequencies, and the mean power on each frequency band was extracted for all epochs. For the theta phase—gamma power intermodulation analysis, the theta phase at 7 Hz was extracted and divided into eight equal sized phase angle bins. The average gamma (20–90 Hz) power corresponding to each theta phase bin was then calculated. Ripple oscillation in the CA1 pyramidal layer were detected using the Kay_ripple_detector method [36] from the ripple_detection package from Eden-Kramer Lab [81].

### Behavioral tests

*Open field* The behavioral apparatus consisted of four 50 cm × 50 cm arenas (light grey PVC, with wall height of 40 cm) placed under camera for tracking the movement of animals by Ethovision XT15 (Noldus). The illumination was applied by indirect diffuse room light (20–25 lx). Each animal was released in one of the corners and monitored for 10 min. The mice were then removed to the holding cage and a 12 cm × 4 cm semi-transparent 50 ml falcon tube was placed in the center of each arena. The animals were then released in the arena and observed for additional 10 min.

*Elevated plus-maze (EPM)* The maze consisted of two open arms (30 × 5 cm) and two enclosed arms (30 × 5 cm, inner diameter) connected by central platform (5 × 5 cm) and elevated to 40 cm above the floor. The floor of each arm was light grey and the closed arms had transparent (15 cm high) side- and end-walls. The illumination level in all arms was ~ 150 lx. The mouse was placed in the center of the maze facing one of the enclosed arms and observed for 5 min. The latency to the first open arm entry, number of open and closed arm entries (four paw criterion) and the time spent in different zones of the maze were measured. The number of faecal boli was counted after trial. Distance travelled and time spent in different areas (open, closed) was recorded with Ethovision XT 10 tracking equipment (Noldus, Netherlands).

*IntelliCage (IC)* Mice were subcutaneously injected with RFID transponders (Planet ID GmbH, Germany) for individual identification. The IntelliCage by NewBehavior (TSE Systems, Germany) is an apparatus designed to fit inside a large cage (610 × 435 × 215 mm, Tecniplast 2000P). The apparatus itself provides four recording chambers that fit into the corners of the housing cage. Access into the chambers is provided via a tubular antenna (50 mm outer and 30 mm inner diameter) reading the transponder codes. The chamber contains two openings of 13 mm diameter (one on the left, one on the right) which give access to drinking bottles. These openings are crossed by photo beams recording nose-pokes of the mice and the holes can be closed by motorized doors. Four triangular red shelters (Tecniplast, Buguggiate, Italy) were placed in the middle of the IntelliCage and used as sleeping quarters and as a stand to reach the food. The floor was covered with a thick (2–3 cm) layer of bedding. The IntelliCage was controlled by a computer with dedicated software, executing preprogrammed experimental schedules and registering the number and duration of visits to the corner chambers, nose-pokes to the door openings and lickings as behavioral measures for each mouse. In the beginning of the test, the mice were released in the IntelliCage with all doors opened allowing unlimited access to the bottles (free adaptation).

*Adaptation to nose-poke* All doors were closed at the beginning of experiment and mice were required to poke into closed gates to reach drinking tubes. Only the first nosepoke of the visit opened the door for 5 s (pre-defined time). Animals had to start a new visit in order to get access to water again.

*Adaptation to drinking sessions* Doors were programmed to open after the first nose-poke only during two 2-h periods, from 20:00 to 22:00 and from 04:00 to 06:00. Drinking sessions were applied for increasing the motivation to visit the corners and thereby providing defined time windows for testing of learning.

*Flexible sequencing task* The animals were assigned two correct corners, which were rewarded alternately during drinking sessions (task acquisition—after visiting a correct corner, the next reward could be obtained in diagonally opposite corner, correct sequence of visits 1–3-1–3 etc., corners 2 and 4 were assigned as incorrect and never rewarded). After 4 days (8 sessions) the sequence was reversed, i.e. reward (water) was delivered in previously incorrect corners for next 8 sessions. After first reversal session, two more reversals were performed.

*Home cage activity* Male Control and *Gad-Grik1*^−/−^ were housed individually in cages with an infrared sensor (InfraMot; TSE-Systems) to monitor their activity over 6 days (excluding the first night of adaptation) with 12/12 h dark/light cycle. The mean hourly activity during the dark/active phase of mice was averaged over the 6 days.

*Barnes maze (BM)* The maze consists of a circular platform (100 cm diameter) with 20 holes (5 cm diameter) around the perimeter (Ugo Basile, Italy). One of the holes was connected with a dark chamber filled with bedding material and two food pellets, the escape box. Two days before the experiment, each animal was introduced to the escape box for 2–3 min. The bright light (500–600 lx on the platform) was used to motivate the mice to find and enter the escape box. The mice were trained to find the escape box in three (day 1–2) or two (day 3) training trails per day (inter-trial interval at least 60 min) over three days. The training trial ended when the mouse entered the escape box or after 3 min as cut-off time (in this case, the mouse was gently directed to the escape box). The memory test was carried out during the first trial on day 4 when the mice were monitored on the platform without escape box for 90 s. Thereafter, reversal learning was carried out, where the escape box was moved under the opposite hole and the mice received two training trials on day 4 and 5. After the last training trial on day 5, the second memory test was performed. Throughout the testing, the movement of animals was tracked by Ethovision XT15 (Noldus).

### Statistical analysis

All data was transferred to GraphPad Prism (v. 9.3.1.471 or v. 9.4.1.681) for statistical analysis. The basal frequency of sIPSCs and of sEPSCs in control and *Gad-Grik1*^−/−^ pyramidal CA3 cells across different age groups (neonatal, juvenile and adult) were analyzed by 2-way ANOVA. To compare the event frequencies between the genotypes within each age group we used multiple Mann–Whitney test as a post-hoc, as the model residuals were not normally distributed. The basal frequencies of the spontaneous network bursts in control vs *Gad-Grik1*^−/−^ neonatal hippocampus were normally distributed and compared by unpaired two-tailed t-test test. For testing of drug effects (application vs baseline), two-tailed paired t-test or Wilcoxon match-pairs sign-rank test were used, for normally or not normally distributed model residuals, respectively. All the statistical tests were performed on raw data. For the graphical representation of the drug effects, the frequency during the drug application was normalized to that during the baseline recording.

The spike frequency, burst duration and frequency, and activity spread clusters in the MEA data were analyzed by 2-way ANOVA. To compare the activity spread during 0–20 ms and 30–70 ms after DG spike we used the Mann–Whitney U-test. For the in vivo data, we used 2-way ANOVA to compare the theta and gamma power, ripple frequencies and durations, and sum-of-squares F-test to test the quadratic curve fit. We used 2-way ANOVA to compare the groups and Holm-Šídák’s test as post-hoc for multiple pairwise comparisons in the behavioral experiments. We used two-tailed unpaired t-test for comparing the relearning slopes calculated for the Barnes Maze experiments.

## Supplementary Information

**Additional file 1: Figure S1.** **A**. Basal amplitude of sEPSCs and sIPSCs in CA3 pyramidal cells from acute control and *Gad-Grik1*^−/−^ slices across different age groups (neonatal: n = 24(17) and 18(13); juvenile: n = 14(12) and 12(10); adult: n = 10(8) and 10(9), for control and *Gad-Grik1*^−/−^ respectively; n refers to number of cells, followed by number of animals in parenthesis. Bars represent mean ± SEM. Amplitudes were compared by 2-way ANOVA. **B**. Effect of ATPA (1μM) on sEPSC and sIPSC amplitude in CA3 pyramidal cells from acute control and *Gad-Grik1*^−/−^ slices at different stages of development (neonatal: n = 14(8) and 10(9); juvenile: n = 10(10) and 8(7); adult: n = 6(6) and 7(7), for control and *Gad-Grik1*^−/−^, respectively). **C**. Effect of ACET (200 nM) on the amplitude of sEPSCs and sIPSCs in neonatal control and *Gad-Grik1*^−/−^ slices (n = 13(10) and 7(6), for control and *Gad-Grik1*^−/−^, respectively). Bars represent mean ± SEM. The amplitude of events is normalized to the baseline, and the amplitude during ATPA / ACET application is compared to the baseline by paired t-test.

**Additional file 2: Figure S2**. **A**. Oscillatory power in the theta frequency range for channels located in the CA3 *stratum moleculare* and CA3 *stratum pyramidale*, for male and female control and *Gad-Grik1*^−/−^ mice (n = 5 / group). **B**. Oscillatory power in the gamma frequency range for channels located in the CA3 *stratum moleculare* and CA3 *stratum pyramidale*, for male and female control and *Gad-Grik1*^−/−^ mice (n = 5 / group). **C**. Oscillatory power in the low theta (3-6 Hz) frequency range separately for epochs of resting or moving in male and female control and *Gad-Grik1*^−/−^ mice. **D**. Oscillatory power in the high theta (7-13 Hz) frequency range separately for epochs of resting or moving in male and female control and *Gad-Grik1*^−/−^ mice. High theta was elevated in the *Gad-Grik1*^*−/−*^ mice were moving, * p = 0.0167, Holm-Šídák posthoc test after 2-way ANOVA.

## Data Availability

The datasets and materials used and/or analyzed during the current study are available from the corresponding author on reasonable request.
