# Supplementary figures and images for: Interneuronal GluK1 kainate receptors control maturation of GABAergic transmission and network synchrony in the hippocampus
Source: Mol Brain. 2023 May 20;16:43. doi: 10.1186/s13041-023-01035-9 (PMC10199616; doi:10.1186/s13041-023-01035-9)

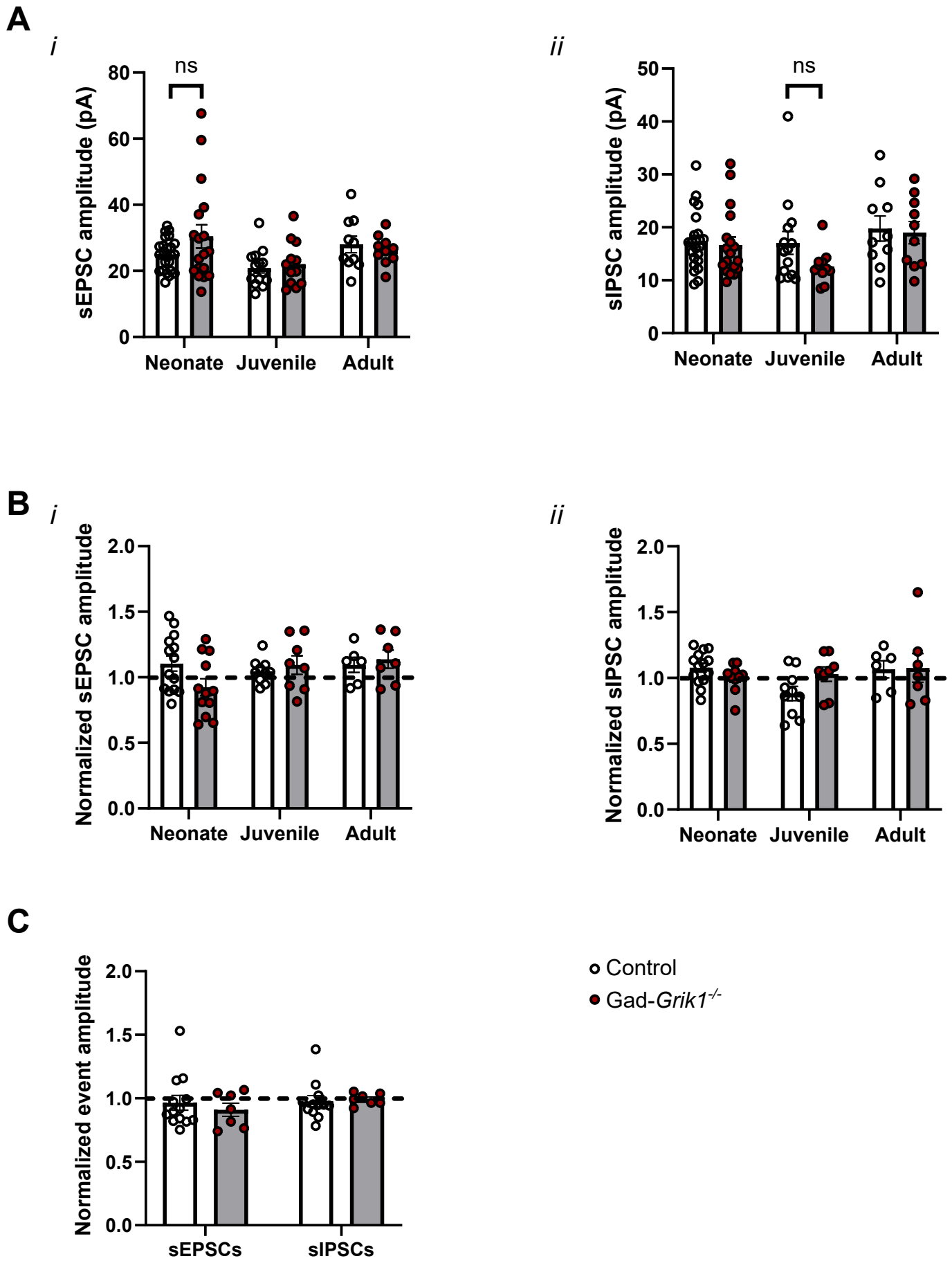

Supplement: Supplementary file 1 — Additional file 1: Figure S1. A. Basal amplitude of sEPSCs and sIPSCs in CA3 pyramidal cells from acute control and Gad-Grik1−/− slices across different age groups (neonatal: n = 24(17) and 18(13); juvenile: n = 14(12) and 12(10); adult: n = 10(8) and 10(9), for control and Gad-Grik1−/− respectively; n refers to number of cells, followed by number of animals in parenthesis. Bars represent mean ± SEM. Amplitudes were compared by 2-way ANOVA. B. Effect of ATPA (1μM) on sEPSC and sIPSC amplitude in CA3 pyramidal cells from acute control and Gad-Grik1−/− slices at different stages of development (neonatal: n = 14(8) and 10(9); juvenile: n = 10(10) and 8(7); adult: n = 6(6) and 7(7), for control and Gad-Grik1−/−, respectively). C. Effect of ACET (200 nM) on the amplitude of sEPSCs and sIPSCs in neonatal control and Gad-Grik1−/− slices (n = 13(10) and 7(6), for control and Gad-Grik1−/−, respectively). Bars represent mean ± SEM. The amplitude of events is normalized to the baseline, and the amplitude during ATPA / ACET application is compared to the baseline by paired t-test. [file 13041_2023_1035_MOESM1_ESM.pdf]

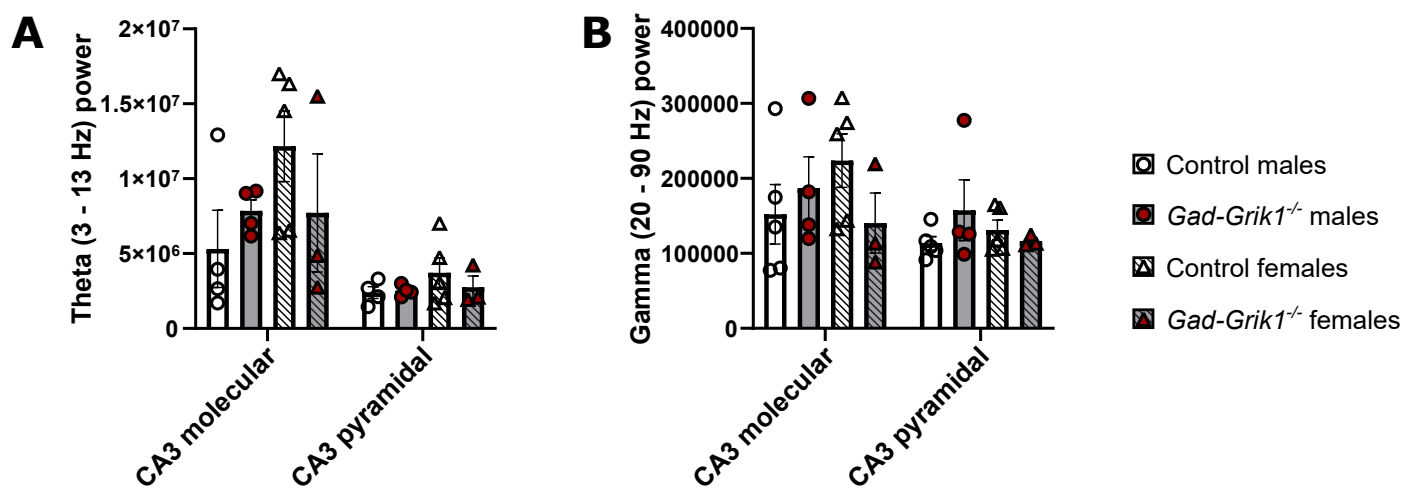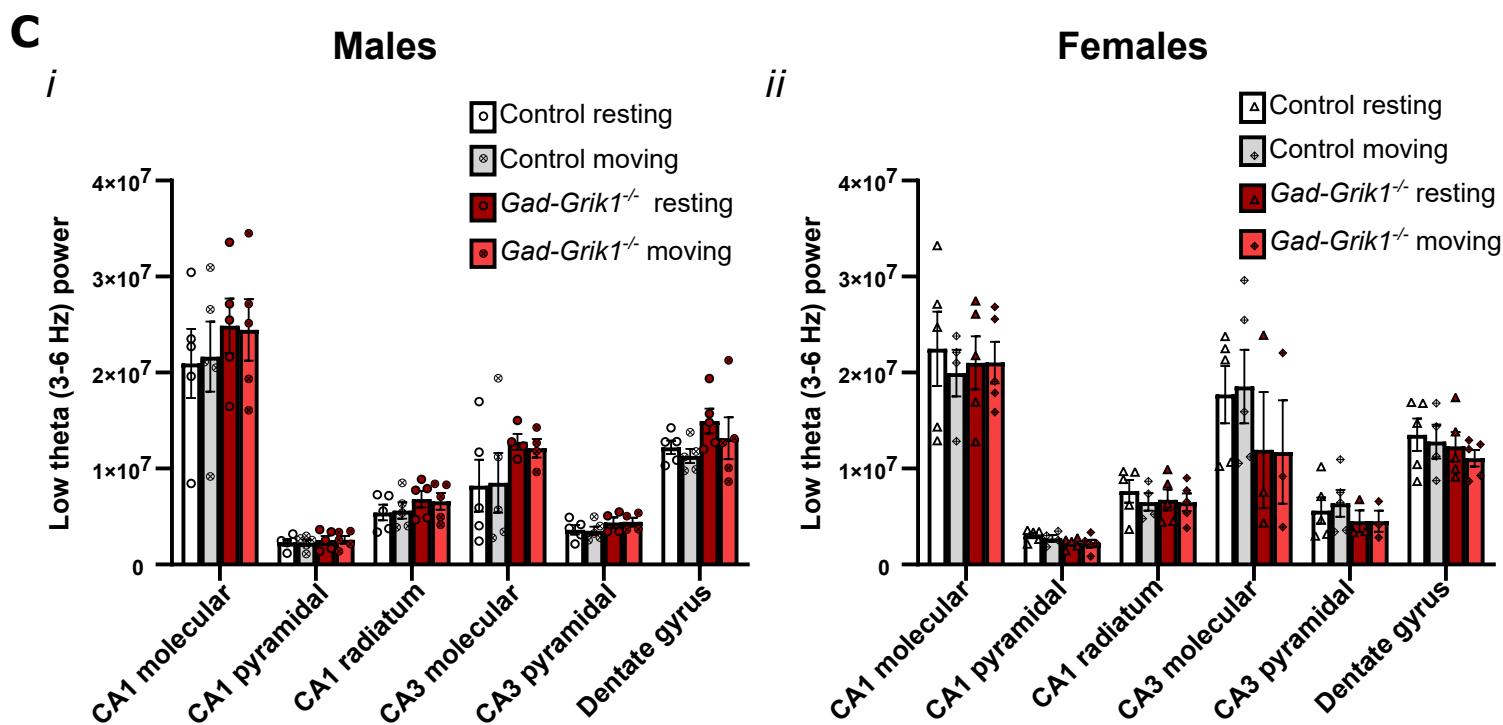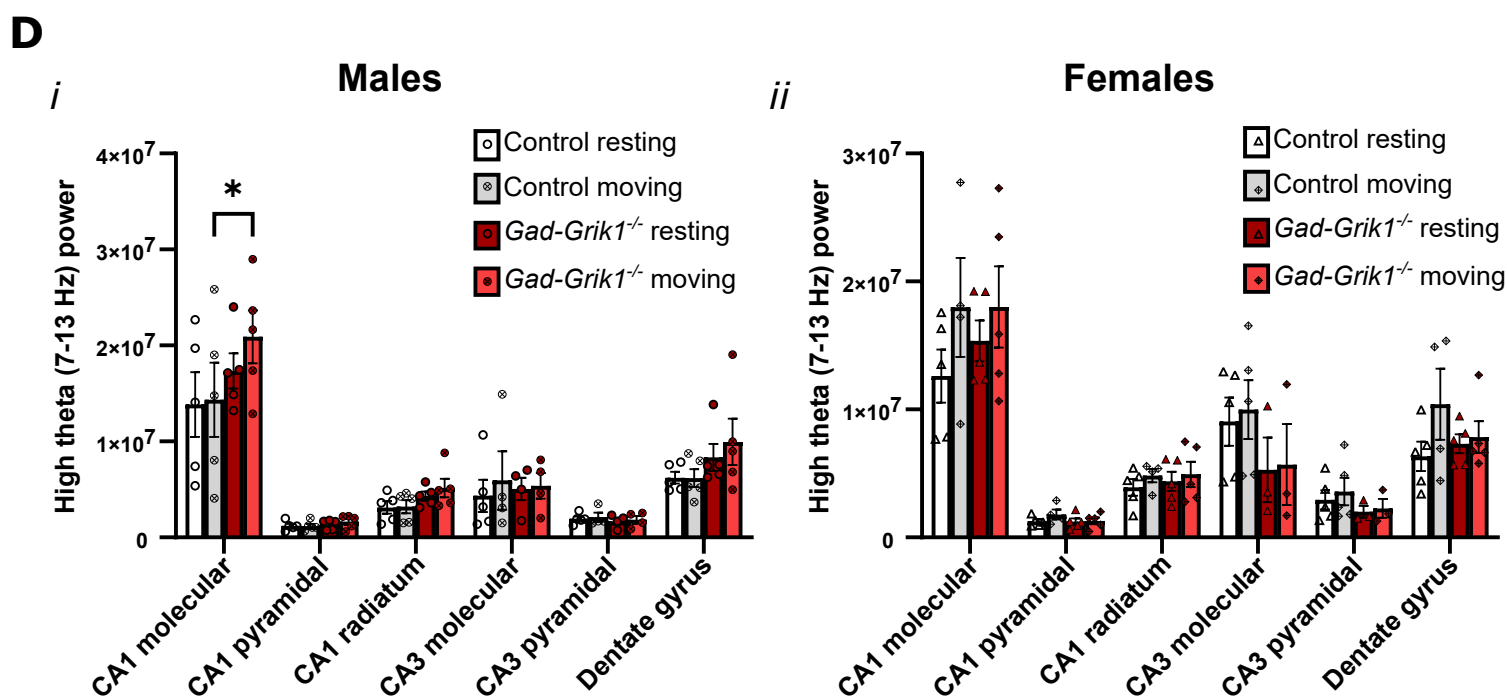

Supplement: Supplementary file 2 — Additional file 2: Figure S2. A. Oscillatory power in the theta frequency range for channels located in the CA3 stratum moleculare and CA3 stratum pyramidale, for male and female control and Gad-Grik1−/− mice (n = 5 / group). B. Oscillatory power in the gamma frequency range for channels located in the CA3 stratum moleculare and CA3 stratum pyramidale, for male and female control and Gad-Grik1−/− mice (n = 5 / group). C. Oscillatory power in the low theta (3-6 Hz) frequency range separately for epochs of resting or moving in male and female control and Gad-Grik1−/− mice. D. Oscillatory power in the high theta (7-13 Hz) frequency range separately for epochs of resting or moving in male and female control and Gad-Grik1−/− mice. High theta was elevated in the Gad-Grik1−/− mice were moving, * p = 0.0167, Holm-Šídák posthoc test after 2-way ANOVA. [file 13041_2023_1035_MOESM2_ESM.pdf]
